# Evidence that Orai1 does not contribute to store-operated TRPC1 channels in vascular smooth muscle cells

**DOI:** 10.1080/19336950.2017.1303025

**Published:** 2017-03-16

**Authors:** Jian Shi, Francesc Miralles, Jean-Pierre Kinet, Lutz Birnbaumer, William A. Large, Anthony P. Albert

**Affiliations:** a Institute of Cardiovascular & Metabolic Medicine, School of Medicine, University of Leeds, Leeds, UK; b Vascular Biology Research Centre, Institute of Molecular & Clinical Sciences Research Institute, St. George's, University of London, Cranmer Terrace, London, UK; c Institute of Medical & Biomedical Education, St. George's, University of London, Cranmer Terrace, London, UK; d Laboratory of Allergy and Immunology, Department of Pathology, Beth Israel Deaconess Medical Center, Harvard Medical School, Boston, MA, USA; e Laboratory of Neurobiology, National Institute of Environmental Health Sciences, Research Triangle Park, NC, USA; f Institute of Biomedical Research (BIOMED), Catholic University of Argentina, Buenos Aires, Argentina

**Keywords:** Orai1, PLC, STIM1, store-operated, TRPC1, vascular smooth muscle

## Abstract

Ca^2+^-permeable store-operated channels (SOCs) mediate Ca^2+^ entry pathways which are involved in many cellular functions such as contraction, growth, and proliferation. Prototypical SOCs are formed of Orai1 proteins and are activated by the endo/sarcoplasmic reticulum Ca^2+^ sensor stromal interaction molecule 1 (STIM1). There is considerable debate about whether canonical transient receptor potential 1 (TRPC1) proteins also form store-operated channels (SOCs), and if they do, is Orai1 involved. We recently showed that stimulation of TRPC1-based SOCs involves store depletion inducing STIM1-evoked Gαq/PLCβ1 activity in contractile vascular smooth muscle cells (VSMCs). Therefore the present work investigates the role of Orai1 in activation of TRPC1-based SOCs in freshly isolated mesenteric artery VSMCs from wild-type (WT) and Orai1^−/−^ mice. Store-operated whole-cell and single channel currents recorded from WT and Orai1^−/−^ VSMCs had similar properties, with relatively linear current-voltage relationships, reversal potentials of about +20mV, unitary conductances of about 2pS, and inhibition by anti-TRPC1 and anti-STIM1 antibodies. In Orai1^−/−^ VSMCs, store depletion induced PLCβ1 activity measured with the fluorescent phosphatidylinositol 4,5-bisphosphate/inositol 1,4,5-trisphosphate biosensor GFP-PLCδ1-PH, which was prevented by knockdown of STIM1. In addition, in Orai1^−/−^ VSMCs, store depletion induced translocation of STIM1 from within the cell to the plasma membrane where it formed STIM1-TRPC1 interactions at discrete puncta-like sites. These findings indicate that activation of TRPC1-based SOCs through a STIM1-activated PLCβ1 pathway are likely to occur independently of Orai1 proteins, providing evidence that TRPC1 channels form genuine SOCs in VSMCs with a contractile phenotype.

## Introduction

Ca^2+^-permeable store-operated channels (SOCs) are physiologically activated by stimulation of the classical phosphoinositol signaling pathway involving Gαq-coupled receptors coupled to inositol 1,4,5-trisphosphate (IP_3_)-mediated depletion of endo/sarcoplasmic reticulum (ER/SR) Ca^2+^ stores. In vascular smooth muscle cells (VSMCs), SOCs mediate Ca^2+^ entry pathways which are proposed to regulate contractility, proliferation and migration, and are considered therapeutic targets for cardiovascular diseases such as hypertension and atherosclerosis.^1-3^ Identifying molecules involved in the composition and activation pathways of SOCs are therefore important objectives in vascular biology.

It is firmly established that prototypical SOCs, termed calcium release-activated channels (CRACs), have properties of high Ca^2+^ permeability, pronounced inward rectification, unitary conductances in the fS range, and are composed of pore-forming Orai1 proteins.^4^ Activation of Orai1-based CRACs is through stromal interaction molecule 1 (STIM1), which senses Ca^2+^ within ER/SR stores and following store depletion undergoes oligomerisation and translocation near to the cytosolic surface of the plasma membrane (PM) where it interacts with Orai1 to induce channel assembly and opening.^4,5^

It is also apparent that many cell types also express SOCs which have very different characteristics to Orai1-based CRACs such as much lower Ca^2+^ permeability, relatively linear rectification, considerably larger unitary conductances, and structures composed of the canonical transient receptor potential family of Ca^2+^-permeable non-selective cation channel proteins (TRPC1-C7).^6^ TRPC1, TRPC3, and TRPC4 channels^6^ have been particularly implicated in composing SOCs, but there are likely to be many distinct TRPC-based SOCs as TRPC subunits can form diverse heteromeric channel structures.^7-9^ Considerable controversy surrounds the legitimacy of TRPC-based SOCs being genuine channels activated by store depletion, since there is uncertainty on whether TRPC subunits compose the conducting pore of SOCs, and how store depletion induces TRPC channel gating. This issue is further supported by TRPC-based SOCs being proposed to be activated or regulated by, or behave as accessory proteins to, STIM1-activated Orai1-based CRAC channels.^6,10-16^

In VSMCs, 2 different SOCs have been described according to cell phenotype. In freshly isolated or primary cultured VSMCs with a contractile phenotype, SOCs exhibit relatively linear rectification, unitary conductances of about 2pS, and are composed of a heteromeric TRPC1/C5 molecular template that may also contain other TRPC subunits.^7-9, 17-20^ As TRPC1 is the essential subunit which confers gating by store depletion these heteromeric channel structures are termed TRPC1-based SOCs.^18-20^ In long-term cultured VSMCs which exhibit a non-contractile and synthetic phenotype,^21^ multiple SOCs have been described with both linear and highly inward rectification, which are reported to involve TRPC1 and Orai1.^22-28^ In both contractile and synthetic VSMCs, STIM1 has been proposed to mediate activation of TRPC1-based and Orai1-based CRACs,^20, 22-25, 28^ which reflects the general consensus that activation by STIM1 is a defining feature of SOCs.^11,12^

Our recent findings have proposed that a STIM1-activated phosphoinositol signaling pathway involving Gαq, PLCβ1, and PKC activities is essential for activation of native TRPC1-based SOCs in contractile VSMCs.^19,20^ Store depletion induces STIM1 to translocate from within the cell to the PM where if forms STIM1-TRPC1 interactions, which stimulate Gαq/PLCβ1 activity to induce PKC-dependent phosphorylation of TRPC1 subunits and channel gating by phosphatidylinositol 4,5-bisphosphate (PIP_2_).^7-9, 17-20, 29,30^ It is not known if Orai1 is involved in this activation pathway, which is an important question if TRPC1-based SOCs are to be considered genuine SOCs. The present study investigates the properties of TRPC1-based SOCs in contractile VSMCs from wild-type (WT) and Orai1^−/−^ mice. We identify that biophysical characteristics and activation mechanisms of TRPC1-based SOCs are unaltered in Orai1^−/−^ VSMCs, providing evidence that the composition and gating of these SOCs are unlikely to require Orai1.

## Results

### Store-operated whole-cell and single channel cation currents in WT and Orai1^−/−^ VSMCs

**Figure 1.**
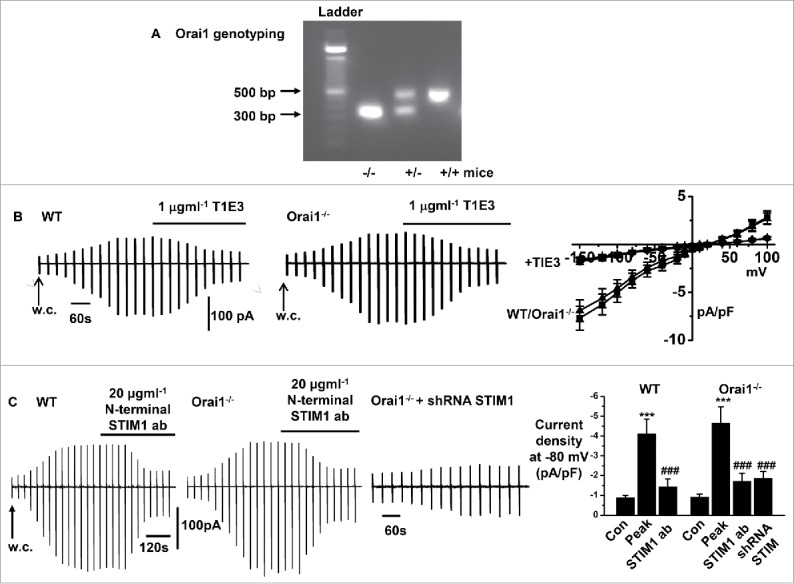
Store-operated whole-cell currents in freshly isolated mesenteric artery VSMCs from WT and Orai1^−/−^ mice A, Analysis of end-point PCR products by agarose gel used to identify Orai1^+/+^, ^+/−^, ^−/−^ mouse genotypes. B, Representative recordings and mean current-voltage relationships showing similar development and peak amplitudes of whole-cell cation currents induced by store-depletion in WT and Orai1^−/−^ VSMCs, which are inhibited by T1E3. C, Representative recordings and mean data showing similar development and amplitudes of whole-cell cation currents induced by store-depletion in WT and Orai1^−/−^ VSMCs, which were inhibited by an externally acting N-terminal anti-STIM1 antibody. In addition, in Orai1^−/−^ cells, transfection with STIM1 shRNA reduced the development and amplitude of mean store-operated whole-cell cation currents. Each point from at least n = 6 patches and n = 3 animals, ***p < 0.001 (control vs. at peak response), ^###^*p* < 0.001 (peak response vs. anti-STIM1 or shRNA STIM1).

In our first experiments, we compared the biophysical properties and involvement of TRPC1 and STIM1 in activation of store-operated whole-cell cation currents in freshly isolated mesenteric artery VSMCs from WT and Orai1^−/−^ mice. Figure 1A shows that end-point PCR products run on an agarose gel were used to identify Orai1^+/+^, ^+/−^, ^−/−^ mouse genotypes as described previously.^31^ Figures 1B & C show that passive depletion of internal Ca^2+^ stores following cell dialysis with a patch pipette solution containing high concentrations of BAPTA and no added Ca^2+^ evoked whole-cell cation currents in both WT and Orai1^−/−^ VSMCs, which had similar mean amplitudes at all membrane potentials tested, relatively linear current-voltage (I/V) relationships, and reversal potentials (E_rev_) of about +20 mV. In addition, Fig. 1B & C illustrate that bath application of T1E3, a TRPC1 antibody which binds to a extracellular pore region of TRPC1 and is known to act as a channel blocker,^7-9, 17-20, 32,33^ and a putative extracellularly-acting N-terminal STIM1 antibody,^20,34, 35^ both inhibited store-operated whole-cell cation currents by over 80% in WT and Orai1^−/−^ VSMCs. Moreover, Fig. 1C demonstrates that in primary cultured Orai1^−/−^ VSMCs, store-operated whole cation currents were greatly reduced by knockdown of STIM1, using a shRNA sequence previously shown to reduce STIM1 expression by over 80%.^20^

**Figure 2.**
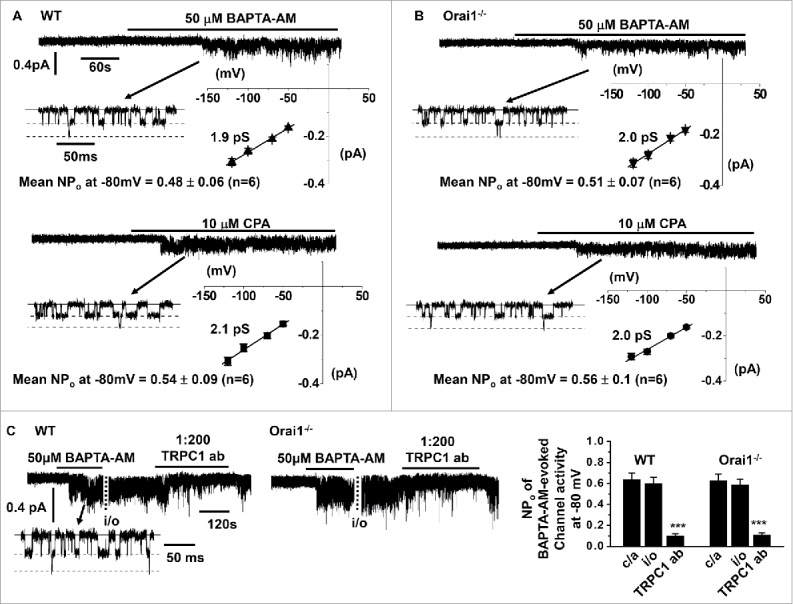
Store-operated single channel currents in freshly isolated mesenteric artery VSMCs from WT and Orai1^−/−^ mice A & B, BAPTA-AM and CPA evoked single cation channel currents with similar mean NP_O_ values in cell-attached patches held at −80 mV, and similar unitary conductances of about 2 pS between −50 mV and −120 mV in WT and Orai1^−/−^ VSMCs. C, Original traces and mean data showing that BAPTA-AM-evoked single channel activity was inhibited by an intracellularly acting anti-TRPC1 antibody in WT and Orai1^−/−^ VSMCs. Each point from at least n = 6 patches and n = 3 animals, ***p < 0.001.

Figure 2A & B show that in WT and Orai1^−/−^ VSMCs, bath application of 1,2-Bis(2-aminophenoxy)ethane-*N,N,N′,N′*-tetraacetic acid acetoxymethyl ester (BAPTA-AM), a cell permeable Ca^2+^ chelator, and the SR Ca^2+^-ATPase inhibitor, cyclopiazonic acid (CPA) evoked single cation channel currents which had similar mean NP_o_ values at −80 mV and unitary conductances between −50 mV and −120 mV of about 2 pS in cell-attached patches. Fig. 2C also shows that BAPTA-AM-evoked cation channel activities, maintained following excision of cell-attached patches into the inside-out configuration, were reduced by about 85% at −80 mV following bath application of an intracellular-acting TRPC1 antibody in WT and Orai1^−/−^ VSMCs.

These results indicate that store depletion induces native TRPC1-based SOCs in contractile VSMCs as described previously in mouse mesenteric arteries and other vascular beds from different animal species,^7-9, 17-20, 29,30^ and confirms the importance of STIM1 in activating these channels.^20^ Importantly, these findings provide evidence that Orai1 is unlikely to have a role in the composition or activation of TRPC1-based SOCs.

### Store depletion-induced PLCβ1 activity in Orai1^−/−^ VSMCs

In a recent study we revealed that store depletion induces interactions between STIM1 and TRPC1, which stimulate PLCβ1 activity required for gating of native TRPC1-based SOCs in contractile mouse and rabbit VSMCs.^19,20^ In the next series of experiments we examined the role of Orai1 in this pathway by monitoring store-operated PLCβ1 activity in primary cultured Orai1^−/−^ VSMCs following transfection with GFP-PLCδ1-PH, a fluorescent biosensor with a high affinity for PIP_2_ and IP_3_^36,37^, and measuring signal changes (measured as relative fluorescent units) at the PM (Fm) and within the cytosol (Fc) as described previously.^19,20^

**Figure 3.**
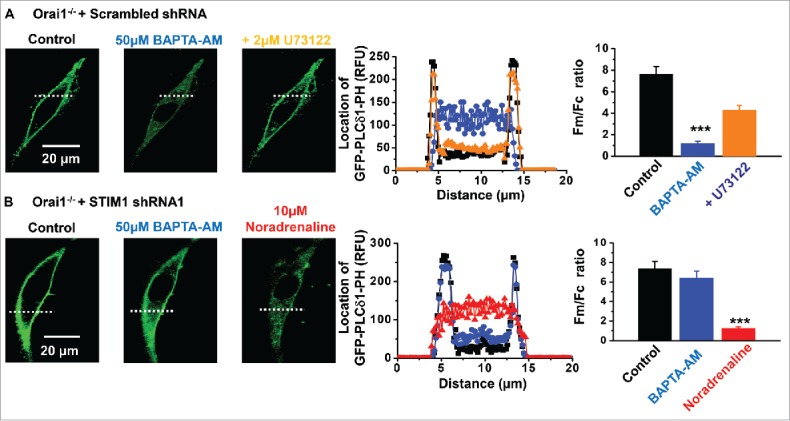
Store-operated PLCβ1 activity in Orai1^−/−^ mice A, In primary cultured Orai1^−/−^ VSMCs transfected with scrambled shRNA (black), BAPTA-AM evoked translocation of GFP-PLCδ1-PH-mediated signals from the PM to the cytosol (blue), which were inhibited by U73122 (orange). B, In Orai1^−/−^ VSMCs transfected with shRNA STIM1 (black), BAPTA-AM-evoked translocation of GFP-PLCδ1-PH signals were reduced (blue). In these cells, noradrenaline (red) stimulated GFP-PLCδ1-PH signals to translocate to from the plasma membrane to the cytosol. Each point from at least n = 20 cells and n = 3 animals, ***p < 0.001.

Figure 3A shows that in Orai1^−/−^ VSMCs, GFP-PLCδ1-PH signals were predominantly expressed at the plasma membrane and had a mean Fm/Fc ratio of about 7, reflecting the predominant cellular location of PIP_2_. Bath application of BAPTA-AM induced translocation of GFP-PLCδ1-PH signals from the plasma membrane to the cytosol with corresponding reductions in mean Fm/Fc ratios, which were inhibited by addition of the PLC inhibitor U73122. These GFP-PLCδ1-PH signal changes represent stimulation of PLCβ1 activity causing PIP_2_ hydrolysis at the plasma membrane and subsequent generation of cytosolic IP_3_.^19,20, 36,37^ Fig. 3B demonstrates that knockdown of STIM1 greatly reduced translocation of GFP-PLCδ1-PH signals induced by BAPTA-AM in Orai1^−/−^ VSMCs. In contrast, stimulation of endogenously expressed α1 Gαq-coupled adrenoreceptors by bath application of noradrenaline induced translocation of GFP-PLCδ1-PH signals from the PM to the cytosol in the presence of STIM1 shRNA, suggesting that STIM1 is not required for PLC activity *per se* as described previously.^20^

These results indicate that store-operated STIM1-evoked PLCβ1 activity, pivotal to stimulation of TRPC1-based SOCs in contractile VSMCs,^19,20^ occurs in the absence of Orai1.

### Store depletion-induced interactions between TRPC1 and STIM1 in Orai1^−/−^ VSMCs

Our studies propose that store-operated interactions between TRPC1 and STIM1 are critical steps in stimulation of Gαq/PLCβ1 activity and activation of TRPC1-based SOCs.^19,20^ We therefore investigated if store-operated TRPC1 and STIM1 interactions occur in Orai1^−/−^ VSMCs.

**Figure 4.**
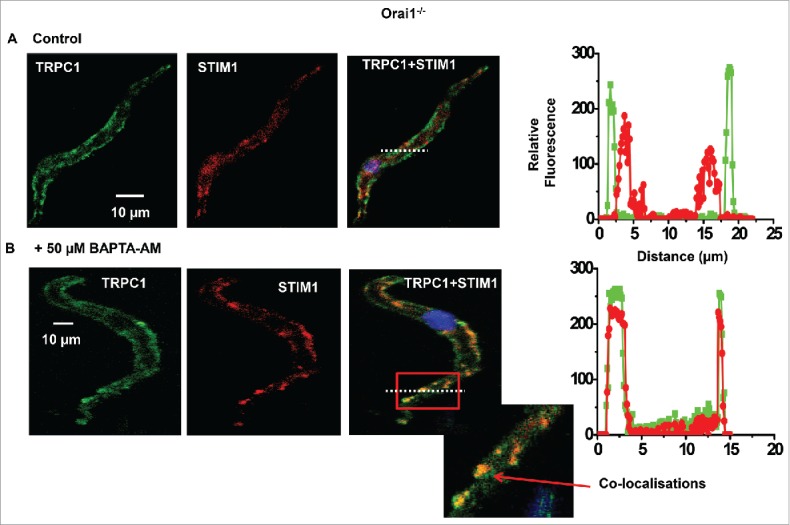
Store-operated interactions between STIM1-TRPC1 in Orai1^−/−^ VSMCs A, Representative images from the same Orai1^−/−^ cell showing staining for TRPC1 (green) and STIM1 (red) were predominantly present at the PM and located within the cytosol respectively. B, In a different Orai1^−/−^ cell treated with BAPTA-AM, both TRPC1 and STIM1 were located at the PM in discrete puncta-like regions. The inset image shows co-localization between TRPC1 and STIM1 staining (yellow) at the PM. Results taken from n = 3 animals.

Figure 4A shows that in freshly isolated un-stimulated Orai1^−/−^ VSMCs, immunocytochemical staining for STIM1 (red) was mainly located within the cytosol whereas staining for TRPC1 (green) was predominantly found at the plasma membrane, and there were few apparent regions of co-localization. Fig. 4B reveal that pre-treatment with BAPTA-AM activated translocation of STIM1 signals from the cytosol to the PM, and also stimulated co-localisations between TRPC1 and STIM1 (yellow) at discrete puncta-like sites. These findings clearly indicate that store depletion stimulates formation of STIM1-TRPC1 complexes at the PM in the absence of Orai1.

## Discussion

TRPC1 channels are ubiquitously expressed among cell types, and are proposed to mediate a wide range of physiologic and pathological functions.^1^ In VSMCs, TRPC1-based SOCs mediate Ca^2+^ entry pathways which contribute to contractility, proliferation, and migration, and are potential therapeutic targets for cardiovascular diseases such as hypertension and atherosclerosis.^1-3^ Therefore understanding the composition and activation mechanisms of TRPC1-based SOCs are important aims.

There are still significant discussions about whether native TRPC-based SOCs exist in physiologic settings including VSMCs, and if Orai1 subunits contribute to the conducting pore or gating mechanism of these channels.^4,6, 10^ The present study investigates these goals in VSMCs with a contractile phenotype, and reports that macroscopic and single channel properties of TRPC1-based SOCs are similar in contractile VSMCs from WT and Orai1^−/−^. Moreover store-operated STIM1-evoked PLCβ1 activity and store depletion-induced interactions between STIM1 and TRPC1 previously shown to be obligatory for activation of TRPC1-based SOCs.^19,20^ are also maintained in Orai1^−/−^ VSMCs. These findings indicate that the composition and activation mechanism of TRPC1-based SOCs in contractile VSMCs are unlikely to require Orai1 subunits.

Our results show that in WT and Orai1^−/−^ VSMCs, well-established store depletion protocols activated whole-cell conductances with relatively linear I/V relationships and an E_rev_ of about +20 mV and single channel currents with a unitary conductance of about 2 pS, which were inhibited by TRPC1 and STIM1 antibodies. These findings confirm previous studies using TRPC1^−/−^ cells and knockdown of STIM1 that functional native TRPC1-based SOCs are expressed in contractile VSMCs and that STIM1 is obligatory for channel opening^7-9, 17-20, 29,30^ In recent pharmacological and molecular studies, we showed that store depletion stimulates interactions between STIM1 and TRPC1 at the PM, which induces Gαq and PLCβ1 activities and PKC-dependent phosphorylation of TRPC1 proteins that is essential for channel opening.^19,20^ Importantly, knockdown of STIM1 and use of TRPC1^−/−^ cells prevented store-operated PLCβ1 activity.^20^ In this study, we show that in Orai1^−/−^ VSMCs store-operated stimulation of PLCβ1 activity recorded using the PIP_2_/IP_3_ biosensor GFP-PLCδ1-PH.^19,20, 36,37^ was reduced by a PLC inhibitor, and prevented by knockdown of STIM1. In agreement with these results, immunocytochemical evidence identified that store depletion stimulated translocation of STIM1 from within the cell to the PM, where it formed STIM1-TRPC1 interactions at discrete puncta-like locations at the PM in Orai1^−/−^ VSMCs. These data reveal that Orai1 subunits are unlikely to contribute to the conducting pore or activation mechanisms involving store-operated STIM1-evoked PLCβ1 activity and store-operated STIM1-TRPC1 interactions of TRPC1-based SOCs in contractile VSMCs.

Orai proteins are composed of 3 subtypes, Orai1, Orai2, and Orai3, which may all form Orai-based CRACs^4,38^ It is therefore possible that Orai2 and Orai3, and not Orai1, may be involved in TRPC1-based SOCs in contractile VSMCs. However, store-operated conductances with characteristics of Orai2- and Orai3-based CRACs such as strong inward rectification and E_rev_ >+50mV.^4,38^ were not revealed in WT and Orai1^−/−^ cells when TRPC1-based SOCs were inhibited with a TRPC1 antibody, or in previous studies performed in TRPC1^−/−^ cells^18,19,20^ It will be important to further investigate possible roles of Orai2 and Orai3 further using knockdown approaches in WT and Orai1^−/−^ VSMCs. In addition, Orai-based CRACs may be present in contractile VSMCs but produce small irresolvable conductances using electrophysiological recordings. A more effective approach might be to investigate if Orai-based Ca^2+^ sparklets, Ca^2+^ entry at localized regions of the PM due to opening of Ca^2+^-permeable channels, are present using total internal reflection fluorescence microscopy.^39^

Store-operated Ca^2+^ entry mediated by TRPC1, Orai1, and STIM1, and a conductance with Orai1-based CRAC channel properties involving activation by STIM1 have been identified in long-term cultured VSMCs,^22-28^ which have a non-contractile, synthetic phenotype that is considerably different from freshly dispersed and primary cultured VSMCs with a contractile phenotype used in the current study.^21^ Taken together, the current evidence indicates that TRPC1-based SOCs are present in contractile VSMCs, whereas TRPC1-based SOCs and Orai1-based CRACs are found in synthetic cells. This is in agreement with low levels of Orai protein expression in contractile VSMCs and much greater expression in synthetic VSMCs.^28,40^ STIM1 activates both TRPC1-based SOCs and Orai1-based CRACs in VSMCs, which supports the proposal that channel gating by store-operated activation of STIM1 is an important criterion for defining SOCs.^11,12^ Selective expression of TRPC1-based SOCs and Orai1-based CRAC channels in distinct VSMC phenotypes may provide useful strategies for developing therapeutic strategies to treat distinct progression phases of cardiovascular diseases such as hypertension and atherosclerosis.

This study provides further evidence that TRPC1-based SOCs are expressed in native cells, and that these channels do not require the presence of Orai1. This provides a strong argument for the existence of multiple SOCs composed of TRPC-based SOCs and Orai-based CRACs, which can function independently of one another.

## Materials and methods

### Reagents

Rabbit anti-TRPC1 antibody (T1E3) was generated by GenScript (Piscataway, NJ, USA) using peptide sequences from a previously characterized putative extracellular region.^1^ Goat anti-TRPC1 (sc-15055), goat anti-STIM1 (sc-79106), and secondary antibodies were obtained from Santa Cruz Biotechnology (Dallas, TX, USA). Rabbit anti-STIM1 antibody against the N-terminal (11565–1-AP) was obtained from Proteintech (Chicago, IL, USA) while mouse anti-GOK/STIM1 (610954) against the N-terminal was obtained from BD Biosciences (Oxford, UK). All other drugs were purchased from Sigma-Aldrich, or Tocris (Abingdon, UK). Agents were dissolved in distilled H_2_O or 0.1% dimethyl sulfoxide (DMSO). DMSO alone had no effect on whole-cell currents or single channel activity.

### Genotyping

This was performed in accordance previous findings.^31^ For each mouse born to Cracm1^+/−^ intercrosses, a 1–2 mm piece of tail was taken and treated with 75 µl lysis solution containing 0.2 mM EDTA and 25 mM NaOH with a pH of 12. The neutralizing solution contained 40 mM Tris-HCl with a pH of 5. Lysed tail-tips were heated at 95^o^C for 1h, cooled to 4^o^C, and then added to 75 µl neutralizing solution. 1 µl of this mixed solution was used as PCR template. The genotyping protocol distinguished WT, Orai1^+/−^, and Orai1^−/−^ in a 3 primer PCR reaction. Primer 1 (5′- TCACGCTTGCTCTCCTCATC-3′) is a forward primer in intron 1, Primer 2 (5′- TAAGGGCGACACGGAAATG-3′) is a forward primer in the genetrap insert, and Primer 3 (5′-AGGTTGTGGACGTTGCTCAC-3′) is a common reverse primer in exon 2. WT mice produced a 488bp band while Orai1^−/−^ mouse produced a 300bp band.

### Cell isolation

WT and Orai1^−/−^ mice were killed using cervical dislocation according to UK Animals Scientific Procedures Act of 1986, and 2^nd^ order mesenteric arteries were dissected free, and cleaned of fat, connective tissue and endothelium in physiologic salt solution containing (mM): 126 NaCl, 6 KCl, 10 glucose, 11 HEPES, 1.2 MgCl_2_ and 1.5 CaCl_2_, pH adjusted to 7.2 using 10 M NaOH. Vessels were enzymatically dispersed into single VSMCs as described previously.^18-20^

### Electrophysiology

Whole-cell and single-channel cation currents were made with an AXOpatch 200B amplifier (Axon Instruments, Union City, CA, USA) at room temperature (20–23°C) as described previously.^18-20^ Whole-cell currents were filtered at 1 kHz (−3dB, low-pass 8-pole Bessel filter, Frequency Devices model LP02, Scensys, Aylesbury, UK) and sampled at 5 kHz (Digidata 1322A and pCLAMP 9.0 software, Molecular Devices, Sunnydale, CA, USA). Whole-cell current/voltage (I/V) relationships were obtained by applying 750 ms duration voltage ramps from +100 to −150 mV every 30 s from a holding potential of 0mV. Single channel currents were filtered between 0.1 kHz and acquired at 1 kHz. Single channel I/V relationships were evaluated by manually altering the holding potential of −80 mV between −120 and +120mV.

Whole-cell recording bath solution contained (mM): 135 mM Na-methanesulfonate, 10 mM CsCl, 1.2 mM MgSO_4_, 10 mM HEPES, 20 mM CaCl_2_, 10 mM glucose, 0.005 mM nicardipine, 0.1 mM 4,4-diisothiocyanostilbene-2,2-disulfonic acid, and 0.1 mM niflumic acid, pH adjusted to 7.4 with NaOH. The patch pipette solution contained 145 mM Cs-methanesulfonate, 20 mM 1,2-bis(2-aminophenoxy)ethane-*N,N,N′,N′*-tetraacetic acid (BAPTA), 8 mM MgCl_2_, and 10 mM HEPES, pH adjusted to 7.2 with CsOH. Under these conditions, voltage-dependent Ca^2+^ channels and Ca^2+^-activated and swell-activated Cl^−^ conductances are blocked allowing cation conductances to be recorded in isolation.

In cell-attached patch experiments the membrane potential was set to 0 mV by perfusing cells in a KCl external solution containing (mM): 126 KCl, 1.5 CaCl_2_, 10 HEPES and 11 glucose, pH adjusted to 7.2 with 10 M KOH. 5 μM Nicardipine was included to prevent smooth muscle cell contraction by blocking Ca^2+^ entry through voltage-gated Ca^2+^ channels. The patch pipette solution used for both cell-attached and inside-out patch recording (extracellular solution) was K^+^ free and contained (mM): 126 NaCl, 1.5 CaCl_2_, 10 HEPES, 11 glucose, 10 TEA, 5 4-AP, 0.0002 iberiotoxin, 0.1 DIDS, 0.1 niflumic acid and 0.005 nicardipine, pH adjusted to 7.2 with NaOH. Inside-out patch bathing solution contained (mM): 18 CsCl, 108 cesium aspartate, 1.2 MgCl_2_, 10 HEPES, 11 glucose, 1 Na_2_ATP, and 0.2 NaGTP, pH adjusted to 7.2 with Tris. Free [Ca^2+^]_i_ was set at 100 nM by adding 0.48 mM CaCl_2_ plus 1 mM 1,2-bis-(2-aminophenoxy)ethane-*N,N,N*′,*N*′-tetraacetic acid(acetoxymethyl ester) BAPTA) using EqCal software (Biosoft, Cambridge, UK).

### Primary cell culture

VSMCs were seeded into culture plates; maintained using DMEM/F-12 media containing 1% serum, and incubated at 37°C in 95%O_2_:5%CO_2_ at 100% humidity for up to 7 d. In 1% serum, VSMCs maintained their contractile phenotype and had similar properties to TRPC1 channel currents in freshly dispersed VSMCs,^19^ which suggest that compensatory changes to channel properties were unlikely in these cell culture conditions.

### Knockdown of STIM1

We used lentiviral-mediated delivery of pLKO.1-puro based shRNA expression plasmids purchased from Sigma-Aldrich to knockdown STIM1 (Gillingham, UK). Infected VSMCs were selected with 2.5 µg/ml puromycin (Invitrogen, San Diego, US) for 2 d before the experiments, and STIM1 levels were determined by Western blotting. STIM1 shRNA sequence used to knockdown STIM1 in mice was 5′-CACCTTCCATGGTGAGGATAA-3′.^20^ Scrambled STIM1 shRNA sequence was used as a control.

### Imaging of GFP-PLCδ-PH-mediated signals

VSMCs were transfected with GFP-PLCδ-PH (Addgene (plasmid ID:21179, Addgene) using Nucleofector™ according to manufacturer's instructions (Amaxa Biosystems, Gaithersburg, MD). 0.2–0.4 μg plasmid DNA was added to 1×10^5^ cells re-suspended in 20 μl Nucleofector™ solution, and cells were kept in primary cell culture conditions for up to 3 d. Transfected cells were imaged using a Zeiss LSM 510 laser scanning confocal microscope and associated software (Carl Zeiss, Jena, Germany). Excitation was produced by 488/405 nm lasers and delivered via a Zeiss Apochromat 63 oil-immersion objective (numerical aperture, 1.4). Two-dimensional images cut horizontally through approximately the middle of the cells were captured (1024×1024 pixels). Final images were produced using PowerPoint (Microsoft XP;Microsoft, Richmond, WA, USA).

### Immunocytochemistry

Freshly isolated VSMCs were fixed with 4% paraformaldehyde (Sigma-Aldrich, Gillingham, UK) for 10 min, washed with phosphate-buffered saline (PBS), and permeabilised with PBS containing 0.1% Triton X-100 for 20 min at room temperature. Cells were incubated with PBS containing 1% bovine serum albumin (BSA) for 1 h at room temperature and then were incubated with primary antibodies in PBS containing 1% BSA overnight at 4°C. The cells were washed and incubated with secondary antibodies conjugated to a fluorescent probe. Unbound secondary antibodies were removed by washing with PBS, and nuclei were labeled with 4′,6-diamidino-2-phenylindole (DAPI) mounting medium (Sigma-Aldrich). Cells were imaged using a Zeiss LSM 510 laser scanning confocal microscope (Carl Zeiss, Jena, Germany). The excitation beam was produced by an argon (488nm) or helium/neon laser (543 nm and 633 nm), and delivered to the specimen via a Zeiss Apochromat X63 oil immersion objective (numerical aperture, 1.4). Emitted fluorescence was captured using LSM 510 software (release 3.2; Carl Zeiss). Two-dimensional images cut horizontally through approximately the middle of the cells were captured (1024×1024 pixels). Raw confocal imaging data were processed and analyzed using Zeiss LSM 510 software. Final images were produced using PowerPoint (Microsoft XP;Microsoft, Richmond, WA, USA).

### Statistical analysis

This was performed using paired (comparing the effects of agents on the same cell) or unpaired (comparing the effects of agents between cells) Student's *t* tests with the level of significance set at a value of *P* < 0.05.
